# An Environmental Data Set for Vector-Borne Disease Modeling and Epidemiology

**DOI:** 10.1371/journal.pone.0094741

**Published:** 2014-04-22

**Authors:** Guillaume Chabot-Couture, Karima Nigmatulina, Philip Eckhoff

**Affiliations:** Institute for Disease Modeling, Intellectual Ventures, Bellevue, Washington, United States of America; Tulane University School of Public Health and Tropical Medicine, United States of America

## Abstract

Understanding the environmental conditions of disease transmission is important in the study of vector-borne diseases. Low- and middle-income countries bear a significant portion of the disease burden; but data about weather conditions in those countries can be sparse and difficult to reconstruct. Here, we describe methods to assemble high-resolution gridded time series data sets of air temperature, relative humidity, land temperature, and rainfall for such areas; and we test these methods on the island of Madagascar. Air temperature and relative humidity were constructed using statistical interpolation of weather station measurements; the resulting median 95^th^ percentile absolute errors were 2.75°C and 16.6%. Missing pixels from the MODIS11 remote sensing land temperature product were estimated using Fourier decomposition and time-series analysis; thus providing an alternative to the 8-day and 30-day aggregated products. The RFE 2.0 remote sensing rainfall estimator was characterized by comparing it with multiple interpolated rainfall products, and we observed significant differences in temporal and spatial heterogeneity relevant to vector-borne disease modeling.

## Introduction

Environmental conditions affect the transmission of vector diseases. The population of a vector depends on the local ecology, and the lifecycle of the disease agent can be modulated by weather variability. Mechanistic factors of disease transmission can appear as correlations between environmental variables and disease incidence; these correlations can in turn be used to describe the geographic distribution of disease risk [1]–[12], early-warning systems [13]–[16], or build mechanistic models of vector population [17]–[21] and disease transmission [22]–[28]. For the purpose of disease modeling and epidemiology, a minimal environmental data set is likely to be composed of air temperature, rainfall, relative humidity, and land temperature.

Air temperature correlates with malaria transmission [29]: when temperature increases, the vector larval development time, the feeding cycle duration, and the parasite maturation time all decrease [26], [30], [31]. Larval mortality also depends on temperature [30], [32]. Air temperature correlates with dengue transmission [33], [34], even though habitat heterogeneity appears to be a confounding factor, and large daily air temperature variations reduce dengue vector survival [32]. Air temperature has also been shown to correlate with the abundance of tsetse flies [5] and with cutaneous leishmaniasis [35].

Rainfall is a well-established correlate of vector abundance for malaria [29], [36]. *Anopheles gambiae* are found mostly in small, temporary habitats [37], while *Anopheles funestus* are found in permanent and semi-permanent rain fed habitats [38]. The survival of *Anopheles gambiae* over the dry season also depends on the level of desiccation reached [18]. In fact, soil moisture calculated from rainfall, land cover, and soil features is a better predictor of biting rates than rainfall alone [39]. An early warning system can be created based on rainfall predictions, either in the short term [16] or in the context of year-to-year oscillations like El Nino/La Nina [40], but extreme rainfall is also linked to larval mortality [41]. Furthermore, rainfall correlates with *Aedes aegyptii* abundance for dengue disease [33], [34], tsetse fly abundance for sleeping sickness [5], and visceral Leishmaniasis [42].

Relative humidity is a determining factor in calculating the rate at which surface water evaporates, and can be used in mechanistic vector habitat models [20], [39]. Relative humidity also affects the survival of vectors differently depending on their species [43], [44]. Overall, using relative humidity as a correlate of vector-borne disease incidence is less common than air temperature or rainfall, but this could change as the complexity of disease and vector models increases.

Land surface temperature has been used as a proxy for air temperature in epidemiological studies of vector-borne diseases [2], [45]–[47]. However, land temperature markedly differs from air temperature during the day and in areas with low vegetation densities [46]. Land surface temperature measurements could also be used to model evapotranspiration [48]–[50] within a mechanistic vector habitat model.

Vector-borne disease dynamics can be extremely heterogeneous in space and time. Environmental data sets with high spatial and temporal resolution are thus needed to accurately model and analyze their relationship with vector-borne diseases. For example, rainfall with daily resolution is probably necessary as aquatic larval stages have a 7–20 day time constant. In space, kilometer resolution enables the capture of rapid changes in land cover, altitude, and it approximately matches the typical mosquito flight distance [51]–[53]. However, it will not be sufficient to accurately represent the small ponds or other habitat features where mosquitoes and flies can multiply [20].

The environment data must also span multiple years: it must be sufficiently long to match the duration of disease incidence measurements [2], to represent the relevant environmental variations, e.g., multi-year oscillations [33], [40], and to span the time scale of interventions, e.g., how long it would take to implement a vaccine or a drug therapy campaign. Furthermore, even mostly constant environmental data layers can be altered in important ways by rapid urbanization, e.g. in Africa, when a multiple-year span is considered [54], [55]; changes in irrigation can create standing water and deforestation can destroy mosquito habitat [56].

Such high-resolution environment data gridded time-series are rare, particularly in developing countries where much of the vector disease burden takes place. Environmental data is most commonly found in the form of monthly climate maps; by comparison, time-series (weather) data over extended regions are rare (IRI/LDEO database [57]). Few products are available “out-of-the-box”, instead requiring significant reformatting or needing to be created through interpolation techniques or patched up to complete missing measurement issues. The availability of data decreases further if spatial resolution must be better than 0.5 degrees of latitude or longitude, and if the temporal resolution must be better than monthly [57]. Specifically for Africa, no gridded time series of air temperature, rainfall, or relative humidity are available at the 30 arc second or 2.5 arc minute resolutions necessary. On the IRI/LDEO database, looking only at products covering the entire African continent, we find the FEWS project [58] which offers daily rainfall with less than 1^o^ grid resolution (0.1^o^), the Aqua satellite MODIS 8-day land surface temperature at 1 km resolution, no gridded daily temperature or daily relative humidity data sets with spatial resolution better than 1^o^.

Assembling a complete environmental data set is a difficult task in Africa and in many areas of the developing world, in part because of the limited availability of ground-based environmental measurements [45]. In these areas of the world, weather stations are few and far between, their locations are biased towards areas of high population density, and many stations have a low reporting frequency. In areas of low weather station density, important inaccuracies can be introduced when air temperature, dew point, or rainfall measurements are interpolated over long distances to the remote areas where much of the vector-borne transmission can take place. Furthermore, other variables like land cover type or vegetation index are not measured by weather stations.

Incorporating remote sensing measurements in the data set can alleviate some of these issues [45]. In the last decades, the spatial, temporal, and spectral resolution of earth-observing satellite instruments have seen large improvements, and the availability and accessibility of remote sensing data sets has also been improving [4], [59]–[61]. However, remote sensing products for near-surface quantities can be acutely limited by line of sight obstructions. For example, near-IR probes, e.g., MODIS, cannot peer through clouds or dense particulates, and microwave probes which can see through clouds can still be affected by rainfall. The orbit of the satellite can also limit the frequency of measurements in a specific location; polar-orbiting satellites cover certain areas around the equator less than twice per day due to the bulging of the earth, and high-resolution satellites can take multiple days to return above a fixed location. The accuracy of remote sensing products can also be limited by the algorithms used to reconstruct the quantity of interest, e.g., air temperature from spectroscopic measurements, or by aspects of the quantity to estimate which are not measured, e.g., orographic precipitation in rainfall estimators.

The reliability of a model or disease map depends on the quality of the underlying data, and on the quality of the model or fit. Input data (on the environment, the vector, or the disease agent) are inherently uncertainty either due to measurement error, aggregation, substitution, or interpolation; even when field validation is extensive [20]. The reliability of a model or disease map can be represented by confidence intervals or assessed through a sensitivity analysis or a validation effort. Most studies only report on their translation of the data or their best fit, without quantifying the limits of their results [1], [3], [4], [11], [18], [62]. Some go further and quantify the confidence intervals from the fitting step, but do not quantify the impact of input data uncertainty [2], [10], [12], [19], [21], [28]. Only a few studies quantify the dependence of their results to input data, through sensitivity analysis or validation [5], [17], [25], [63].

The error of interpolated or aggregated data sets is typically quantified by cross-validation [64], [65], while error on remote sensing estimates is calculated from distributed point measurements, and comparison with other remote sensing products [66]–[71]. Both techniques produce location-independent error distribution assessments. However, the accuracy of such data sets can change significantly in space and time [72], [73]. Accurately quantifying the error in the input data is thus a key component of a sensitivity analysis or of a validation exercise [72], [74].

First, we present the input data and the methods used to construct the air temperature, relative humidity, land surface temperature, and rainfall data sets. Then, we describe the accuracy, the range of validity, and some characteristics of the constructed data sets. Last, we compare our constructed data sets with similar remote sensing products or interpolated products.

## Methods

### Air temperature

**Figure 1:**
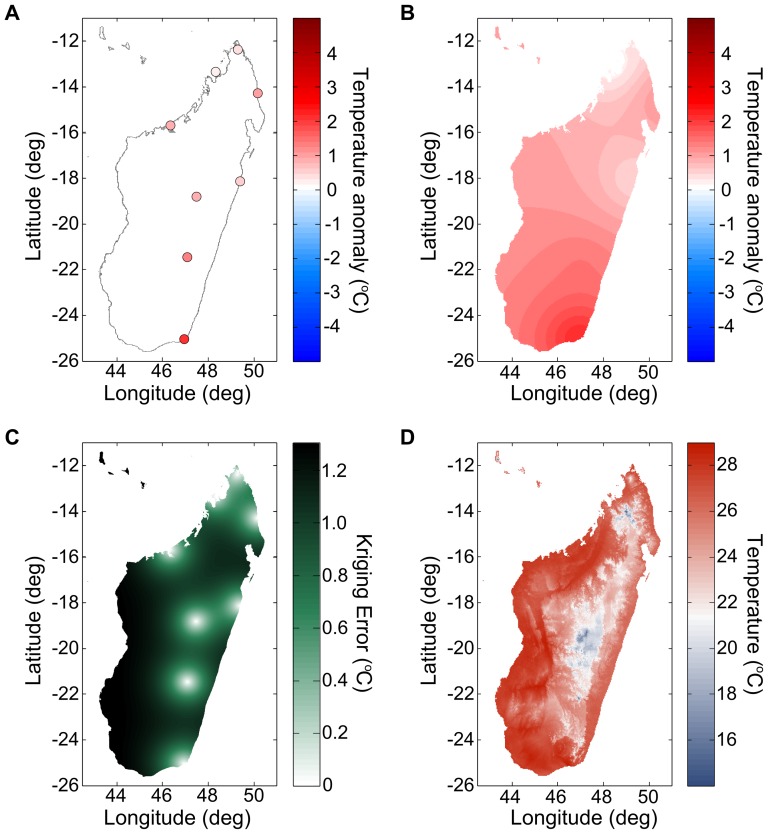
Kriging air temperature from Madagascar weather stations. (A) Extracted air temperature anomalies on January 1^st^, 2010. The periodic seasonal components are removed using Fourier transforms. (B) Kriged anomalies across the island and the resulting error estimate (C). The Kriging error increases away from reporting weather stations at the half-correlation distance speed up to the square root of the sill. (D) Combining the WorldClim-derived average temperature surface for that day and the Kriged temperature anomaly, we obtain a prediction of the air temperature throughout the island. In this last image and throughout the data set, the systematical effects of altitude dominate the day-to-day variations due to weather.

We interpolated weather station measurements of air temperature and dew point taken from the Global Summary Of the Day (GSOD) database [75] using simple Kriging [76]. The GSOD database compiles daily surface weather data from more than 9000 stations, dating in some cases back to 1929. It is available free of charge for non-commercial use. We used simple Kriging to interpolate temperature anomalies from weather station measurements within the region surrounding them. Simple Kriging was used because the mean value of the temperature anomaly is zero, and the form of the distance-dependence in the covariance can be determined from the ensemble of weather stations used. This technique, as opposed to more common distance-based methods [77], can compensate for strongly inhomogeneous weather station distributions and also provides an estimate of interpolation error. We present an example of the entire procedure in Figure 1.

We used the following criteria to effectively reject weathers stations with poor reporting while ensuring that seasonal variations can be extracted: a weather station had to report at least 61 valid measurements within one 365-day window at any point during the station's lifetime, and then, within a histogram of the station's reporting frequency by day-of-the-year (DOY) (e.g., March 2^nd^ 2001 and March 2^nd^ 1937 are both day of year 61), no more than 30 consecutive days could have zero frequency (wrapping around at year end). The longest window of measurements missing from all the years in the data set had to be no longer than 30 days.

**Figure 2:**
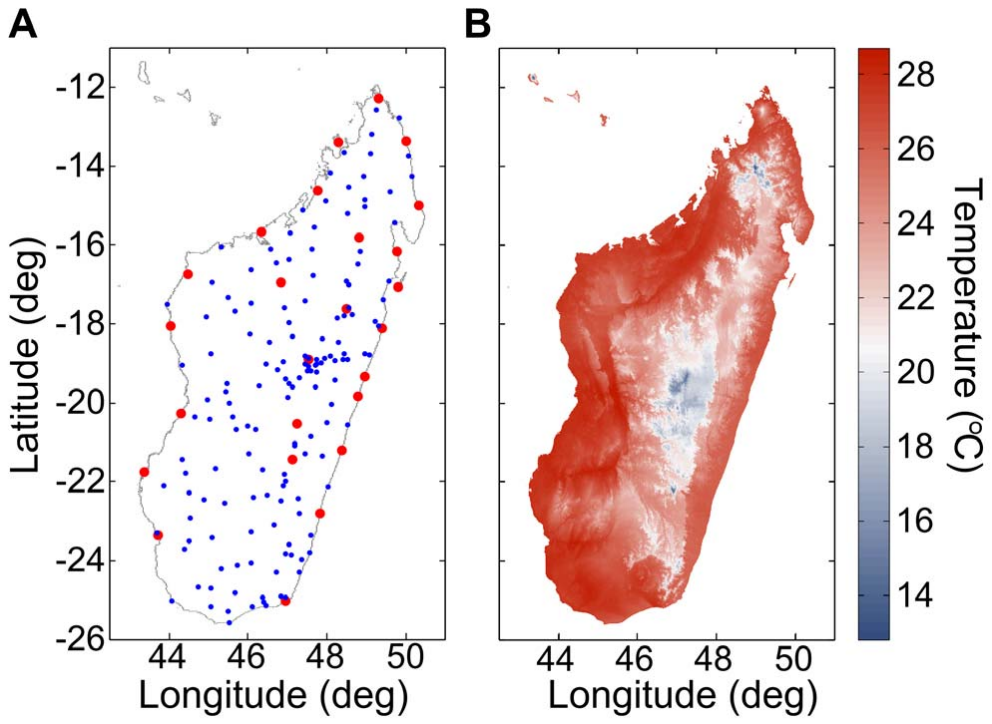
Madagascar climate layer. (A) Weather stations described by Oldeman et al. [108]. The larger red dots are synoptic stations, while the blue dots are simpler field stations. (B) The WorldClim monthly average temperature for January, interpolated from these weather stations [64].

We constructed air temperature by adding a climate layer to spatially interpolated day-to-day temperature anomalies (the weather). We created daily-resolution climate normal maps by temporally interpolating the monthly high-resolution WorldClim data set [64] using the 0-, 1-, 2-, and 3-fold yearly oscillation components of a Fourier decomposition. The WorldClim is a set of global climate layers (climate grids) with a spatial resolution of about 1 square kilometer. It is based on significantly more weather stations than are publicly available, and thus should capture more accurately the systematic effects of geography, e.g., the variations in environmental lapse rate. The published average difference between the spline interpolated surface of this climate data set and weather station measurements (the climate layer error) is less than 1°C [64]. In Figure 2a, we illustrate this difference in Madagascar; the WorldClim data set includes all the weather stations shown in red and blue while the GSOD database includes only a subset of the red (synoptic) weather stations. We present an example climate normal map in Figure 2b.

We calculated the air temperature day-to-day variability across a region using weather stations point measurements and simple Kriging to interpolate between them. For each weather station, we separated the periodic seasonal component from the temperature anomalies by Fourier subtraction of the constant, once-, twice-, and thrice-yearly Fourier components. Since the time series contains many missing or erroneous data, we orthogonalized the Fourier harmonics over the valid measurements in the time series. We present the specifics of the orthogonalization algorithm in File S1. We note that the seasonal signal extracted by this method may be different from the WorldClim climate layer.

**Figure 3:**
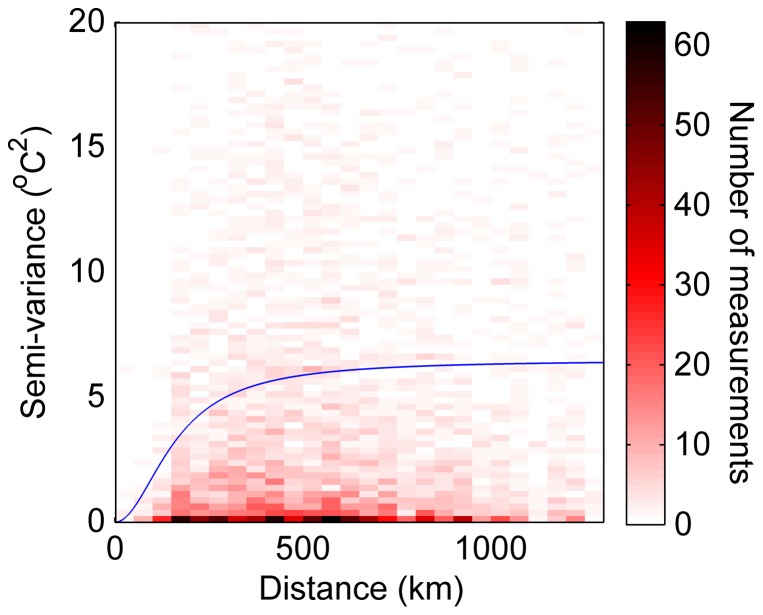
Example variogram. Semi-variance of weather station air temperature measurements over Madagascar (1981–2010), for a given day-of-year. The blue curve is the resulting fit of the functional form presented in the Methods section.

Kriging uses a distance kernel, the semi-variogram, to assign interpolation weights to different weather station measurements [76]. Since the variability and half-correlation distance changes with the time of year, we fit these semi-variograms parameters independently for each day-of-year using
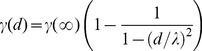
(M1)where γ(∞) is the sill, λ is the half-correlation distance, and *d* is the distance. In our fit, we included all the compiled (distance, anomaly-difference squared) pairs for a specific day-of-year in the 30 years of weather station data considered. In Figure 3, we present an example semi-variogram for January 1^st^ using Madagascar weather stations.

**Figure 4:**
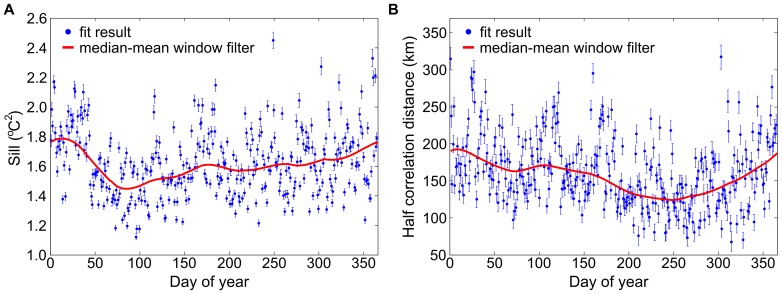
Variogram parameters. Air temperature variogram parameters for Madagascar, from 1981–2010: sill (A) and half correlation distance (B). The blue circles result from fitting the variogram for each day-of-year; the red curve is the smoothed output of the median-mean window filter described in the Methods section and used in our Kriging algorithm.

We smoothed the resulting fit parameters using two filtering passes, first by taking the median in a 31-day sliding window, and second by taking its average also in a 31-day sliding window, before assigning the resulting value to the middle of the window (see Figure 4a and 4b). All elements were set to have the same weight within the averaging window.

While not shown in the formula M1, a nugget effect can be included if it is found that neighboring weather stations are reporting incongruent air temperature values on the same day or if measurement error is believed to be an issue. A nugget effect is a non-zero intercept in the semi-variogram which allows short-range variability in the measured quantity. In the present case, measurement error or local heating effects could create such short-range variability in reported air temperature. When the semi-variogram does not reflect the presence of such short range variability, the Kriging algorithm can become numerically unstable and sometimes produce interpolated values significantly under or over-shooting all measured values.

### Relative humidity

**Figure 5:**
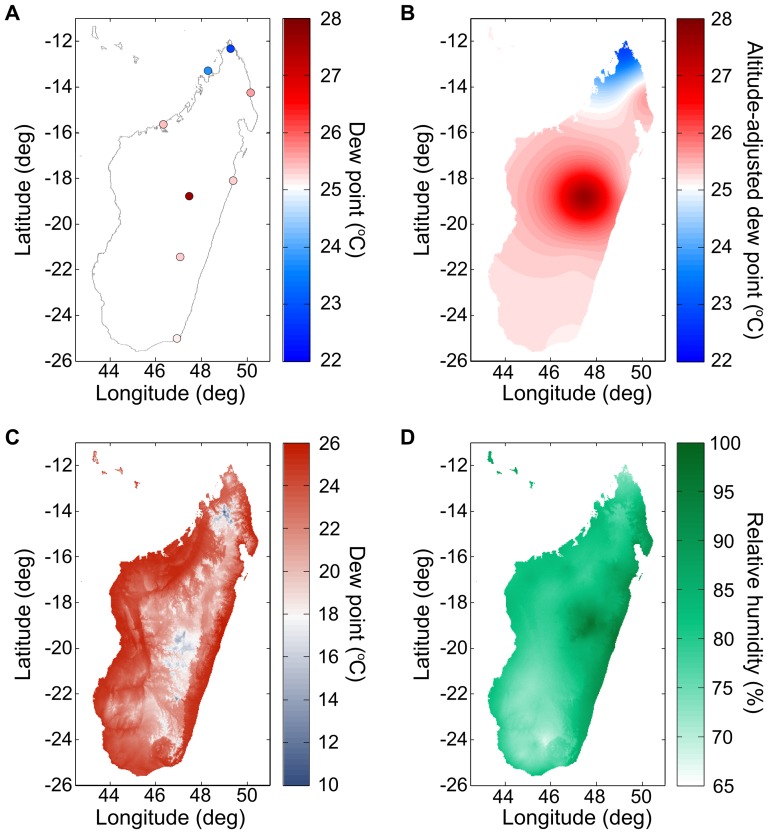
Calculating relative humidity across Madagascar. (A) Weather station dew point measurements on January 1^st^ 2010 (before correcting for altitude). Data from GSOD database [75].(B) Kriged zero-altitude-equivalent dew point values across the island. (C) Altitude-adjusted Kriged dew point. (D) Combining the Kriged air temperature surface with the dew point surface, to obtain the relative humidity throughout the island.

We calculated local relative humidity (RH) by combining maps of air temperature (*T*) and dew point (*T_d_*) using the following formula:



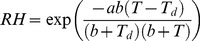
(M2)where a = 17.271, and b = 237.7°C. This formula is based on the August-Roche-Magnus approximation for the saturation vapor pressure of water in air. We constructed maps of air temperature using the algorithm presented in the previous section. We constructed maps of dew point using a modification of the above algorithm, as explained below. In Figure 5, we present the steps in our algorithm to calculate relative humidity using Madagascar as a test case.

**Figure 6:**
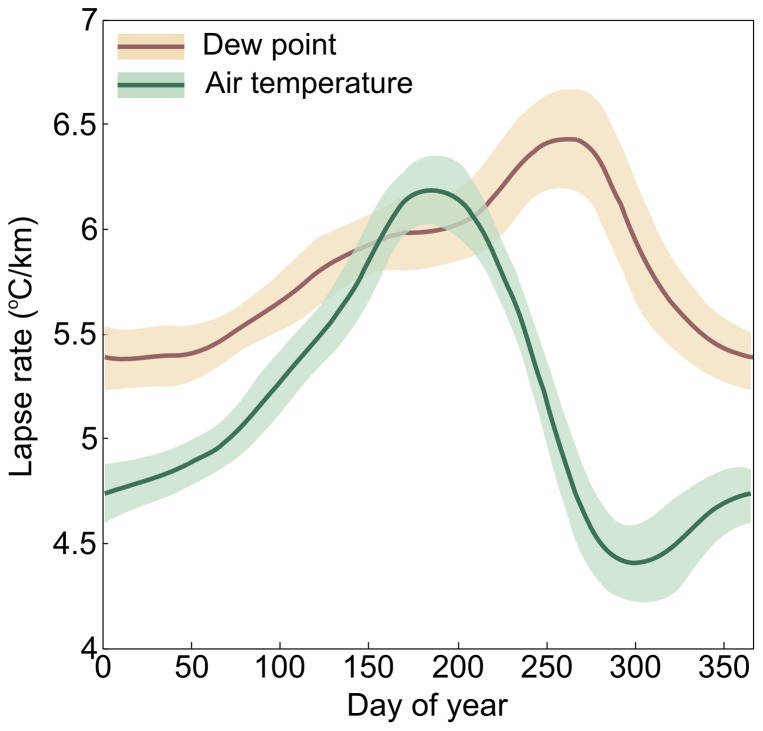
Lapse rates. Dew point and air temperature lapse rates in Madagascar, by day of year. The shaded regions represent one standard deviation above and below the median-mean window filter average curve. Note, only the dew point lapse rate is used in the humidity interpolation algorithm.

Without a high-resolution climate layer on which to base our dew point interpolation, we calculated an approximate climate layer by deriving a region-specific lapse rate for dew point, as a function of day-of-year, using 30-years' worth of (altitude, dew point) pairs (see Figure 6). We smoothed the day-of-year dependence of the lapse rate using two filtering passes: first by taking the median in a 31-day periodic window, and second by taking the average of the result also in a 31-day periodic window. In the present case, the region is the island of Madagascar; a region will be of appropriate size if it contains a sufficient number of weather stations without being so large as to encompass very different climates.

**Figure 7:**
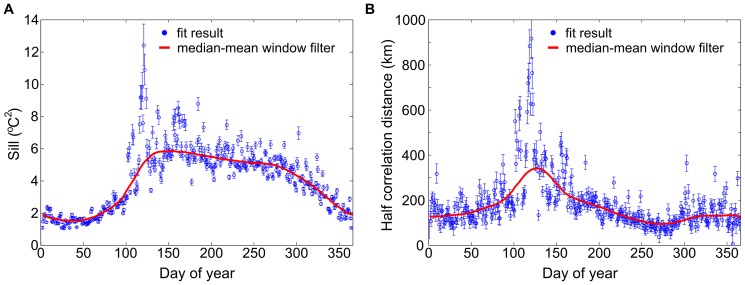
Variogram parameters. Dew point variogram parameters for Madagascar, from 1981–2010, by day-of-year: sill (A) and half correlation distance (B). The blue circles result from fitting the variogram for each day-of-year; the red curve is the smooth output of the median-mean window filter described in the Methods section and used in our Kriging algorithm.

Using this lapse rate, we corrected for (subtracted) the effect of altitude in dew point measurements (Figure 5a) to obtain their zero-altitude equivalent. We then Kriged [76] these zero-altitude dew-point equivalents in order to obtain a zero-altitude dew point map of the region (Figure 5b). We derived the dew point semi-variogram needed for Kriging as was described in the air temperature section (Figures 7a–7b). Finally, we re-introduced (added) the effect of altitude using the region-wide lapse rate using an altitude map for the region (Figure 5c). The result is the completed map of dew point needed to calculate relative humidity (Figure 5d).

**Figure 8:**
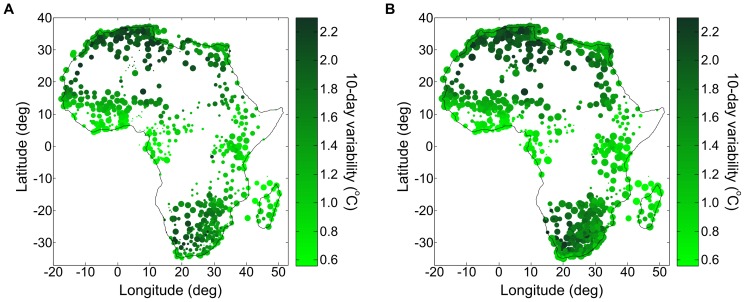
Air temperature variability at weather stations across Africa. Map of the operating weather stations in Africa included in the GSOD database [75], within (A) the 1981–2000 period and (B) within the 2001–2010 period. Each weather station is represented by a filled circle. Its size is proportional to its reporting frequency (maximum size corresponds to daily reporting), and its color corresponds to the 10-day air-temperature variability. Certain regions of Africa have a dense network of reliable weather stations (e.g. South Africa) while other regions are simply devoid of weather stations (e.g. DRC). Air temperature variability is smallest at the equator (around 0.5°C) and increases up to 2.5°C at 30 degrees of latitude.

Throughout Africa (including the island of Madagascar), 923 out of a total of 1403 weather stations met our quality criteria (as described in the air temperature method section) for air temperature in the 1981–2011 period [75]. For dew point measurements, 914 out of 1403 met these criteria. Since the reliable weather stations reporting dew point are essentially the same as those reporting air temperature, their spatial distributions are equivalent (see Figure 8). As a result, the accuracy of air temperature and the accuracy of relative humidity will be strongly correlated.

### Land temperature

Land temperature was derived from spectral radiance measurement of the MODIS instrument aboard the AQUA satellite. Twice a day this satellite passes over a point on the earth's surface at approximately 1:30am and 1:30pm local solar time. However, measurement of the ground temperature is not always valid as, for example, the satellite view angle can be obscured by clouds or heavy aerosol. We here describe an algorithm to estimate these missing measurements.

Land surface temperature measurements were first acquired from NASA in raw tiles. We used the MYD11A1 v005 product [78]. The MODIS land surface temperature product has been validated, and is accurate to 1°C (but better than 0.5°C in most cases) [66]–[68]. These tiles were then projected to a 30-arc-second WGS84 latitude/longitude grid. The time series for each latitude/longitude pair in the grid (pixel of the image) was then Fourier analyzed. First, we filtered out the noise in the signal due to the satellite repeat cycle by removing harmonics with 16-day periodicity as well as other noise frequencies which stood high above the aperiodic signal level. Then, we extracted the seasonality of the land temperature by measuring the 0-, 1-, 2-, and 3-fold yearly oscillation amplitudes, and we kept it for further processing. The standard Fourier transform procedure was modified to deal with the missing values, see File S1 for details.

After we removed the noise and seasonality from the raw signal, the daily land temperature anomalies remained. We stationarized this time series by dividing it by its seasonally-varying standard deviation. We measured the standard deviation as a function of day of year using a 31-day weighted window (the weights are w(t) = 1-|t/16|^3^). This gave us, for each pixel, a time series of constant zero mean and constant unit standard deviation, albeit with missing values. Within the valid values of this time series, outliers were defined as any measurement four standard deviations above or below the mean. If any outliers are found, e.g., a freezing day during the summer, we removed them (they became missing values) and recalculate the mean and standard deviation of the time series until no more could be found. We refer to this final time series as the normalized-departure time series.

Pathological pixels were identified as was done for weather stations in the air temperature methods: if a pixel contained more than 30 consecutive days of the year without measurements, or contained less than 61 valid measurements in any 365 day period, they were removed. Because we required a mean and standard deviation at every point, for all days of the year, we used the average of valid adjacent points to obtain a mean and a standard deviation for pathological pixels. In some cases, a pathological pixel did not have adjacent valid pixels. In those cases, and only if the extent of the pathological regions is small (2–3 pixels in diameter), we completed the pathological pixel iteratively, one by one, starting with those which had the most number of adjacent valid pixels. Using this method, isolated pathological pixels were eventually connected to valid pixels through interpolated pixels. If a pathological pixel was an island, without any neighboring valid pixels, a reasonable value was used in its place, e.g., the nearest land average. For the period between 2002 and 2011, this method was successful in Madagascar and Nigeria, but failed in India due to the continuous cloud cover over large regions during the monsoon.

**Figure 9:**
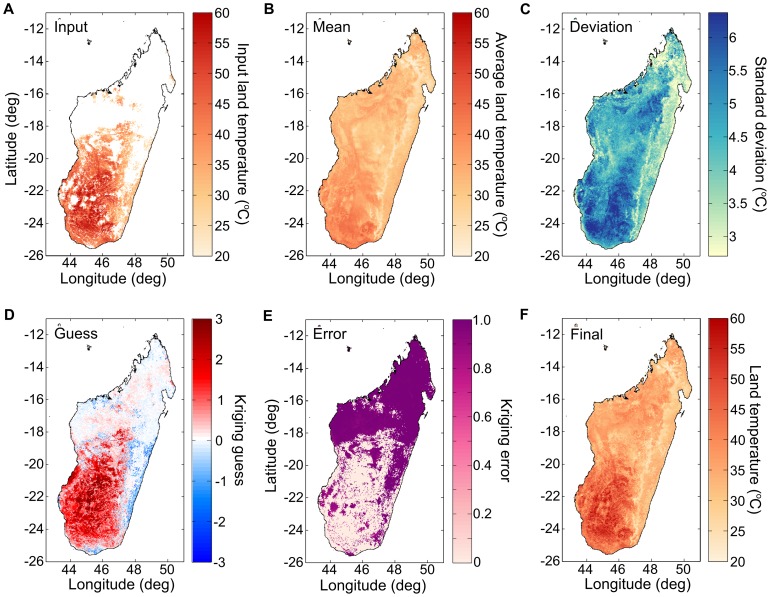
Land temperature surface completion method steps. (A) Remote-sensing measurements of land temperature contain invalid and/or missing pixels (shown in white). The measurements shown here are from the MYD 11A1v005 data set [78]. In order to estimate the land temperate at these missing pixels, the algorithm first calculates the land temperature average (B) and standard deviation (C), for each pixel, for that day of year. At each pixel, the temporal Kriging algorithm then produce a Kriging guesses (D) and a Kriging error (E). Combined with the average of the valid land temperature pixels for that day, a final land temperature surface is constructed (F).

The result is that the land temperature data set is composed, at each pixel, of a normalized departure time series, as well as an average temperature (MEAN) and a standard deviation (STD) for each day of the year (see Figure 9).

Since there is some day-to-day temporal correlation in the land temperature measurements, we adapted the simple Kriging technique to interpolate valid measurements in time [76]. First, we calculated the autocorrelation function for each pixel and then fit their time delay dependence to an analytical form in order to construct the covariance matrix and the covariance vector. A simple power law provided a good and robust fit across all pixels. The measured autocorrelations were fitted only up to the smallest lag, which had a negative autocorrelation value. This maximum useful lag was used as the maximum distance over which to look for valid measurements to include into the Kriging prediction. For example, if the autocorrelation function was only valid up to a lag of five days, then a valid measurement six or more days before or after the missing value was not included in the Kriging calculation. If the autocorrelation function fit failed due to poor statistics, then Kriging was not performed on that pixel and the uncertainty of all the missing values on that specific pixel were not reduced.

Using this autocorrelation function and the valid measurements within the maximum lag, a covariance matrix and a covariance vector were constructed around each missing measurement. From there, simple Kriging returned a best mean (the Kriging guess) and the size of the remaining (unknown) variance (the Kriging error).

Once all the missing values had been treated as outlined above, information from valid measurements in the same pixel but at different times was factored into the estimate, but not information from valid measurements at the same time but in different pixels. In principle, it could have been possible to calculate a covariance function between measurements distant in space but equal in time, and to use Kriging in order to optimally estimate the value of a stochastic field from a few nearby measurements. In the present case however, performing an exact Kriging calculation for all the missing points within a country, day by day, for up to 10 years would wave been very computationally intensive. Instead, we estimated the average land temperature anomaly on that calendar day (A_avg_), in the region of interest, and linearly combine it with the time-derived best guess (G) using the remaining variance (E) not accounted for in the time-based estimate as weight. By multiplying this normalized departure estimate with the standard deviation and average land surface temperature, for that pixel and day, we obtained our final estimate for the land surface temperature (LST) (see example in Figure 9): 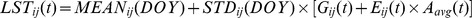
Here, the subscripts *ij* represent the latitude/longitude index within the grid; they index the pixels. This formula does not account for the cooling effect of clouds during the day or their warming effect at night. One possible way to include this effect could be to additively incorporate the cloud flag into the estimate of the anomaly.

### Rainfall

We interpolated the RFE 2.0 [58] data set from a 0.1 degree to a 30 arc seconds spatial resolution using bi-linear interpolation. As suggested by the release notes, we replaced any value exceeding 300 mm/day with 300 mm/day and we replaced missing values with 0 mm/day.

The RFE 2.0 rainfall estimator is available in Africa starting January 1^st^ 2001 [58] which is based upon work by Xie and Arkin [79]. It has also been calculated for parts of the middle-east and south Asia. It combines remote-sensing measurements from the AMSU-A and -B sensors onboard NOAA satellites, the SSM\I and SSM\IS sensors on board DMSP satellites, and the infrared imagers onboard the METEOSAT satellites. AMSU-B and SSM\I are microwave sounders, they estimate rainfall by measuring the amount of upwelling microwave scattered radiation from ice particles in the air [80]. These measurements are available up to four times a day and have a horizontal resolution around 30 km at nadir. The METEOSAT satellites estimate the rain rate using the cloud-top temperature measured by infrared images [81]. These images are available every half hour, with a horizontal resolution around 4 km.

Cross-validation of RFE 2.0 product shows it has a 50% correlation with measurements on the ground and a small negative bias of -0.15 mm/day [82]. Amongst the methods available to estimate rainfall in regions of low weather station density, the RFE 2.0 product has sufficiently high spatial and temporal resolution and shows excellent performance [15], [83]. The MiRS rainfall estimator also combines different satellite measurements, but it has only been archived since August 30^th^, 2007 [84].

## Results

### Air temperature

We measure the accuracy and precision of our interpolation method using cross-validation, i.e., comparing the true value measured at a weather station, with the interpolated value calculated without measurements from that weather station. The computed average bias and the prediction variance can be compared with the computed Kriging error. We repeat this procedure for each weather station.

**Figure 10:**
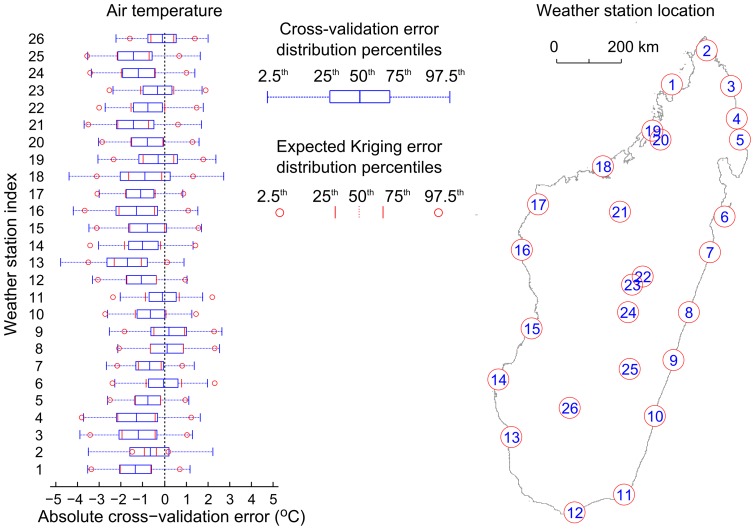
Cross-validation of air temperature Kriging estimates across Madagascar. (Left) For each weather station, the observed absolute error distribution (blue) is compared with the median error distribution predicted by the Kriging interpolation method (red). The percentiles corresponding to the different features of the boxplot are explained in the legend. (Right) Index of weather station location in Madagascar.

For air temperature interpolation, the resulting error distributions are shown in Figure 10, and compared with what is predicted by the Kriging algorithm. The median daily bias is −0.786°C, the median daily mean absolute error is 1.21°C, the median daily root mean squared error is 1.47°C, and the median daily 95^th^ percentile absolute error is 2.75°C. Here, and systematically in what follows, the median is taken across the 26 weather stations on Madagascar. Computing 10-day error measures is more appropriate due to the natural time scale of vector-borne diseases. As such, the median 10-day bias is −0.436°C and the median 10-day 95^th^ percentile absolute error is 1.49°C.

The predicted air temperature values are significantly biased, with the true value lying outside of the interquartile prediction range for 15 out of 26 weather stations. This is due to the underlying WorldClim climate layer which is used to offset the Kriging predictions for unmeasured points. This observed bias (−0.786°C) is consistent with the reported average error of this climate layer (less than 1°C) [64]. This bias-related interpolation error could possibly be reduced by substituting the WorldClim climate layer by a lapse-rate corrected temperature surface, as is done for dew point interpolation.

The width of the central mass of the error distribution is similar to the Gaussian distribution assumed by the Kriging method. This can be seen by the good agreement between the predicted and observed inter-quartile distance (25^th^ to 75^th^ percentile). By contrast, the tails of the observed error distribution are significantly more dispersed and more asymmetrical than for a Gaussian distribution; this can be seen by comparing the location of the 2.5^th^ and 97.5^th^ percentile markers in Figure 10. In File S1, detailed air temperature predictions and Kriging error distributions are presented for each weather station.

Spatial interpolation error is maximal far from weather stations, and can be estimated from the maximum Kriging error (black regions in Fig. 1c). For a given distribution of weather stations, the Kriging error is proportional to the temporal and spatial variability of air temperature. For a point multiple spatial correlation distances away from any weather stations, the prediction error reduces to the regional temperature variability added to the climate layer error. We can use this result to estimate the maximum Kriging error across Africa.

In Figure 8, we present maps of the operating weather stations across Africa, first for the 1981–2010 period (A) and then for the 2001–2010 period (B). Each dot represents one weather station which passes our quality criterion (described in the Methods section). The area of each filled circle is proportional to its reporting frequency (the largest dots represent stations that report at least once a day), and the color of the filled circle corresponds to its 10-day temperature variability. The number of reporting stations has decreased in the last ten years, but the remaining stations have increased their reporting frequency. There are extended regions in Africa for which no weather station measurements are available, e.g., in the Democratic Republic of the Congo (DRC). However, in the DRC the average combined error is approximately 1.5°C because the 10-day variability of air temperature is low near the equator. By comparison, in northern Algeria the average combined standard error is approximately 2.5°C. Interestingly, while the 10-day temperature variability increases away from the equator, the density of weather stations also increases; the resulting net change (increase or decrease) in Kriging error will depend on the correlation length of air temperature.

### Relative humidity

As above, we evaluate the accuracy and precision of our relative humidity interpolation algorithm by cross-validation. Since relative humidity is calculated using dew point and air temperature, we present cross-validation results first for dew point, and then for relative humidity. The interpolation error of relative humidity is calculated assuming that the errors and biases that affect dew point and air temperature are uncorrelated.

**Figure 11:**
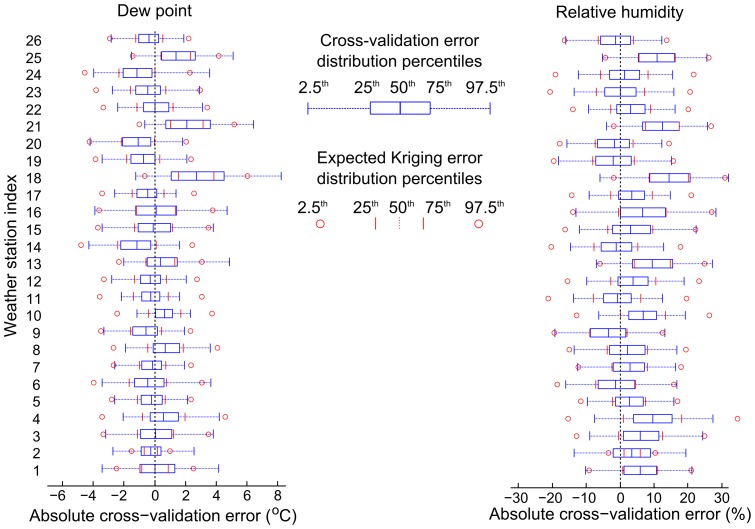
Cross-validation for relative humidity Kriging estimates across Madagascar. (Left) cross-validation error distributions for dew point, and (right) cross-validation errors for relative humidity. The percentiles corresponding to the different features of the boxplot are explained in the legend.

Dew point cross-validation normalized error distributions are shown in Figure 11a, and compared with what the Kriging method predicts. The median (across weather stations) dew point daily bias is −0.177°C, the median mean absolute error is 1.25°C, the median RMSE is 1.64°C, and the median 95^th^ percentile absolute error is 3.32°C. Computing 10-day error measures is more appropriate due to the natural time scale of vector-borne diseases; as such, the median dew point 10-day bias is −0.289°C and the median dew point 10-day 95^th^ percentile absolute error is 2.27°C. Dew point predictions have less bias error than air temperature predictions do: the true value is outside of the interquartile prediction range for only 6 out of the 26 weather stations. Further study of the large bias observed for stations 18, 21, and 25 may help to improve the interpolation algorithm. Both the predicted interquartile range and the 95% confidence intervals agree well with what the Kriging method anticipates.

Relative humidity cross-validation normalized error distributions are shown in Figure 11b, and compared with what the Kriging method predicts. The median (across weather stations) relative humidity daily bias is 3.09% (percentage points), the median mean absolute error is 6.61%, the median RMSE is 8.38%, and the median 95^th^ percentile absolute error is 16.6%. Computing 10-day error measures is more appropriate due to the natural time scale of vector-borne diseases; as such, the median relative humidity 10-day bias is 3.08% (percentage points) and the median 10-day 95^th^ percentile absolute error is 11.0%. Since the air temperature was systematically more underestimated by the Kriging algorithm than dew point, the resulting relative humidity is systematically overestimated: the true value is outside of the interquartile prediction range for 8 out of the 26 weather stations. In File S1, detailed predictions and Kriging error distributions of dew point and relative humidity are presented for each weather station.

**Figure 12:**
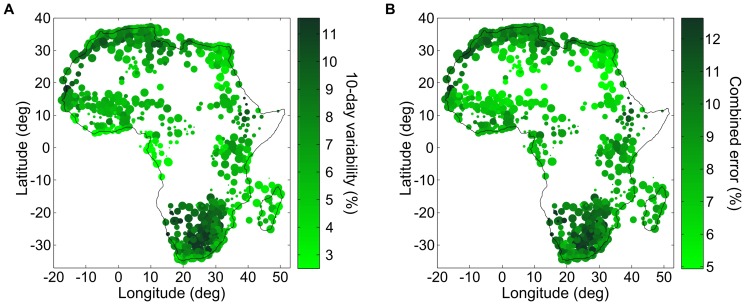
Relative humidity, 10-day variability, and combined error. (A) 10-day variability of relative humidity reported by weather stations across Africa (from GSOD database [75]), averaged over the 2001–2010 reporting period. Size of filled circles proportional to the reporting frequency of the station; 10-day variability on a green color scale. (B) By combining the 10-day variability and climate layer error, we obtain a combined average error on relative humidity that is indicative of what the average maximum Kriging error would be.

In our algorithm, the error of relative humidity is the combination of the Kriging (interpolation) error and the supporting climate layer error. The climate layer error for air temperature is that of the WorldClim data set (less than 1°C), and the climate layer error for the dew point is related to the lapse rate fit. The Kriging error is at most the intrinsic variability of the data set. In Figure 12a, we present the average 10-day variability of the relative humidity for weather stations across Africa. In Figure 12b, we combine this variability taking a fixed climate layer error of 1°C for both dew point and air temperature in order to estimate what average maximum error of our interpolation algorithm could produce in other regions. We note that the largest driver of error is the variability of the relative humidity. The combined standard error, which varies between 5% and 12.5%, is applicable only far from weather stations. In regions of higher weather station density, the climate layer and Kriging errors will decrease. The relative humidity in coastal areas varies less than the in-land relative humidity.

In our Madagascar example (Figure 6), we observe that the environmental dew point lapse rate is season dependent (as expected [85]), and is larger than the air temperature lapse rate, except during the dry season. This should be contrasted with the moist adiabatic lapse rate, which is typically smaller than the environmental lapse rate, and corresponds to the rate at which a “parcel of air”, saturated with moisture, will cool when it rises. The altitude at which the air temperature equals the dew point and moisture starts to condense is called cloud base. By comparison, the environmental dew point lapse rate we calculate includes the effect of orographic precipitation of the average climate in the region. In this case, the relative humidity tends to decrease with altitude, except during part of the dry season.

The interpolated dew point can in some situations be greater than the interpolated air temperature. When that is the case, the interpolated relative humidity will be greater than 100%; the user of the algorithm may then cap the calculated value to 100%, or use it as an indication that precipitation may have taken place in that area.

### Land temperature

We have tested our method in three countries: Madagascar, Nigeria, and India. Our method can limit the number of pixels for which it is not possible to determine, by Fourier transform, a mean temperature for all days of the year; we refer to them as pathological pixels. These pixels are completed using neighboring valid pixels. For Madagascar and Nigeria, we were able to complete missing all pixels using this method. In the southern region of India however, our method fails due to the presence of large, contiguous areas of pathological pixels resulting from the Monsoon.

**Figure 13:**
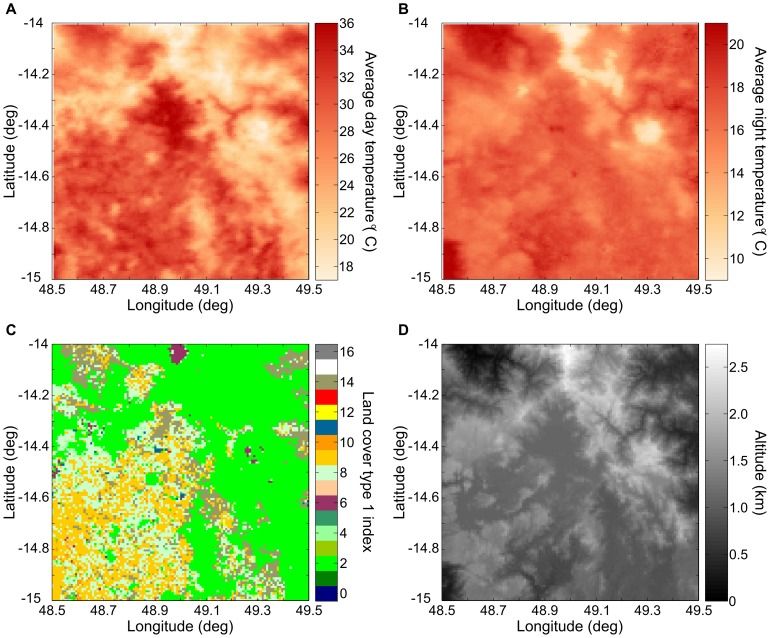
Daytime and nighttime land surface temperature. (A–B) Average land surface temperature in a region of Madagascar for January 1^st^ during the day (at approximately 2 pm) and at night (approximately 2 am) from MODIS aboard the AQUA satellite. (C) Land cover type 1 index classification from the MODIS 12 product [78]. The dominant land cover classification in this region are evergreen broadleaf forest (index 2), savannas (index 9), woody savannas (index 8), barren or sparsely vegetated (index 16), and closed shrubland (index 6). (D) Altitude of the region from the WorldClim data set [64]. Daytime land surface temperature correlates with land cover type; nighttime land surface temperature correlates with altitude.

During the day, land surface temperature correlates with land cover type. In a region of Madagascar, Figure 13a shows the average daytime land surface temperature for January 1^st^ and Figure 13b shows the MODIS Type 1 land cover product. The demarcation between high and low land surface temperature corresponds to the demarcation between low and high-density forest canopy areas. By comparison, this correlation is weaker for nighttime measurements. At night, the land surface temperature correlates with altitude in the same way that air temperature does. In the same region of Madagascar, Figure 13c shows the average nighttime land surface temperature for January 1^st^ and Figure 13d shows altitude. While land cover type and altitude are correlated in this region, nighttime land surface temperature measurements are more correlated with altitude than they are with land cover type.

### Rainfall

**Figure 14:**
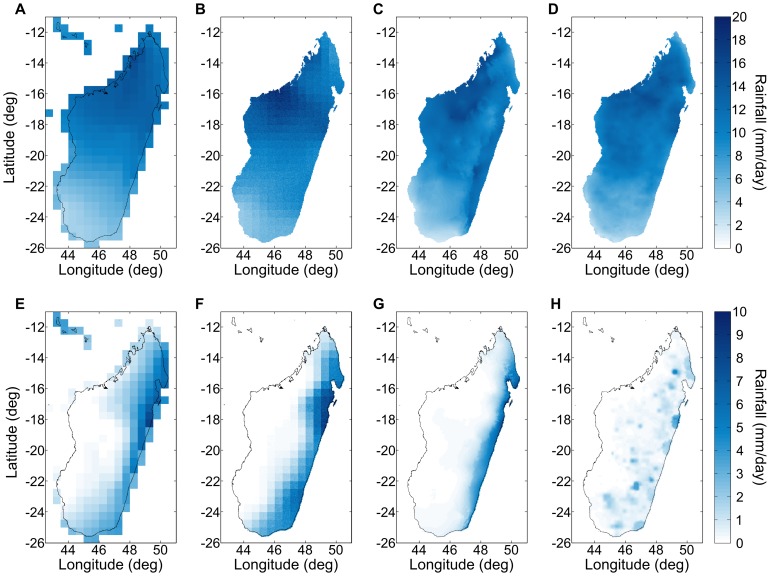
A comparison of rainfall climate layers. Average January rainfall across Madagascar according to (A) the CRU2.1 CL (1991–2000) data set [88], (B) the GPCC (1995–2004) data set [89], and (C) the WorldClim (1950–2000) data set [64] compared to (D) the average rainfall from the RFE 2.0 (2001–2010) data set [58]. Average July rainfall according to (E) the CRU2.1 CL (1991–2000) data set [88], (F) the GPCC (1995–2004) data set, and (G) the WorldClim (1950–2000) data set compared to (H) the average rainfall from the RFE 2.0 (2001–2010) data set. There is good agreement between the RFE 2.0 derived climate layer and other established climate layers during the rainy season (e.g., January), but during the dry season (e.g., July) all four climate layers differ, the RFE 2.0 most significantly.

Comparing the rainfall climate layer derived from the RFE 2.0 data set to other climate layers, e.g., CRU 2.1 [86]–[88], GPCC [89], and WorldClim [64], can also provide information on the RFE 2.0 accuracy. For example, in Figure 14, we show that all four climate layers are consistent with each other during the rainy season (January) in Madagascar. There are some differences among the interpolated climate layers either due to different weather stations being used or different interpolation methods, but the RFE 2.0 climate layer compares very well with all three. During the dry season (July) however, all four climate layers differ, the RFE 2.0 most significantly. The WorldClim average rainfall is much more limited to low altitude regions on the east coast of Madagascar than the GPCC and CRU 2.1 climate layers because it includes both latitude and altitude as interpolation covariates. By comparison, the RFE 2.0 climate layer shows signs that 10 years are not sufficient to average over the inhomogeneity of rainfall. Nonetheless, further support for the RFE 2.0 data set comes from its good agreement with the GPI+GTS satellite climate layer which use data over a much longer period (1982–2010) [82].

**Figure 15:**
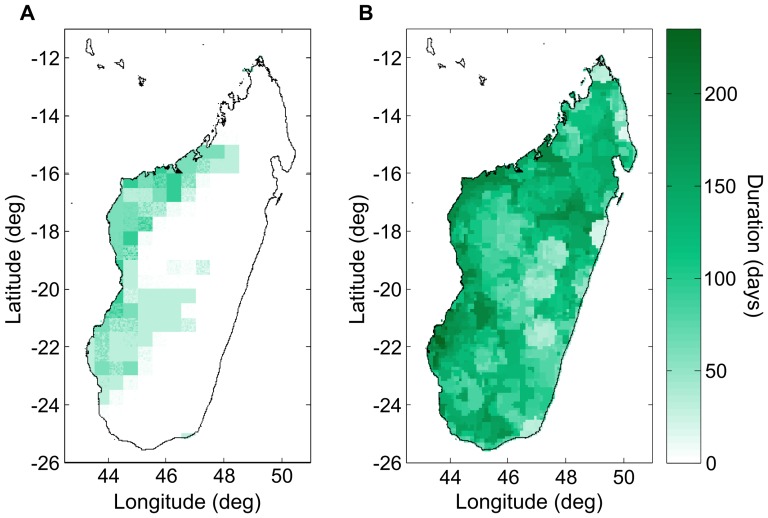
Longest dry spell during a 10-year period. The (A) GPCC (1995–2004) data set [89] contains significantly shorter dry periods than the (B) RFE 2.0 (2001–2010) data set [58]. In the GPCC time series, any rainfall recorded during a 24 hour period at a weather station extends in the neighboring region (due to spatial interpolation), thus effectively reducing the probability of rain-free day.

The length of periods of drought are important in modeling vector-borne diseases because vector habitat is commonly rain-fed or rain-dependent, i.e., puddles, rice fields, river banks, etc. In Figure 15, we compare the longest dry period of the GPCC interpolated rainfall time series with the longest dry period in the RFE 2.0. We see that the RFE 2.0 contains significantly longer dry periods. In the GPCC time series, because it results from spatial interpolation of weather station measurements, any weather station recorded rainfall during a 24 hour period effectively extends in the neighboring region and reduces the probability of a rain-free day.

## Discussion

### Air temperature

Kriging can compensate for the inhomogeneous distribution of weather stations and their bias towards populated areas. Kriging estimates the spatial distribution interpolation error, it compensate for the inhomogeneous distribution of weather stations (e.g., their bias towards populated areas), and it can take into account measurement error and inhomogeneity at short length scales. However, Kriging can be numerically unstable and is more technically complex to implement. By comparison, spline or distance-based methods are simpler and provide good accuracy, but they cannot estimate the spatial or temporal dependence of interpolation error, opting instead to report the median (or average) cross-validation error.

An earlier comparison of the accuracy of remote sensing and spatial interpolation of air temperature data set had shown spatial interpolation to be more accurate [90]. However, a new comparison is warranted to evaluate the performance of spatial Kriging and of novel satellite instruments which measure air temperature directly. Multiple weather satellites are currently capable of estimating atmospheric temperature as a function of altitude. The horizontal resolution of these instruments is typically on the order of 50 km (AIRS, AMSU-A, IASI) [91], which is a fraction of the half-correlation distance of air temperature in the tropics [92], and significantly smaller than the distances between weather stations in Africa (typically 100–300 km). Some instruments have horizontal resolutions down to 5 km, e.g., MODIS 7, but their products are experimental [69], [70], [93], [94] and their time span can be short (2–5 years) which limits their use for modeling. For AMSU+HIRS, the root mean squared error (RMSE) error on surface temperature is 3°C [70]. For MODIS 7, the RMSE on surface temperature is below 4°C [93]. By comparison, the median RMSE of Kriged air temperature was 1.47°C over Madagascar. Overall, spatial interpolation of weather station measurements is an accurate and reliable method to obtain air temperature, even in Africa. In Madagascar, the low density of weather stations does limit the interpolation accuracy, but it has significantly better accuracy than remote sensing products.

In some parts of the world, it is possible to use the land surface temperature as a proxy for surface air temperature. In the Italian Alps, for example, linear relations were established between air temperature measured at weather stations and land temperature measured using a satellite. These linear relations were then used to convert remote sensing measurements of land surface temperature away from weather stations to estimates of the surface air temperature with a RMSE of 1.9°C [95]. Similarly, a statistical model was used to estimate air temperature in Portugal using land surface temperature measurements from MODIS with a RMSE of 1.8°C [63]. In Africa however, a similar analysis was performed in four different countries and a robust relationship between air and land temperature could only be obtained during the night [46], when air and land temperature are more closely related.

### Relative humidity

Relative humidity can be calculated by combining measurements of air temperature and dew point. In some contexts, The interpolation of relative humidity between weather stations is typically performed by individually interpolating air temperature and dew point measurements, and then combining them [96], [97]; a direct interpolation of relative humidity is more difficult [98]. In some contexts, when measurements of dew point are not available, the minimum air temperature during the day can be an acceptable proxy [99].

Few examples of dew point interpolation are available for comparison. Kim et al. [96], using a distance-based interpolation method, quote a mean absolute interpolation error for dew point of 1.3°C; this is comparable to our median mean absolute error of 1.25°C.

A possible improvement to the interpolation of the dew point would be to develop a supporting dew point climate layer that includes all weather stations (not only the ones reporting on a specific day). Alternatively, remote sensing measurements of water vapor profile from MODIS 7 could also be used to create a relative humidity data set with improved spatial resolution; recent work has shown that an accurate relative humidity data set can be obtained in this way [69], [94], [100].

### Land temperature

Cloud cover or heavy aerosol in the satellite line-of-sight can lead to erroneous measurement of land surface radiance. Instrument failure, relief obstructing the line-of-sight, or the bulging of the earth around the equator can also create large regions with missing measurements in remote-sensing data sets. To limit their impact, NASA offers 8-day and 30-day averages of their remote sensing measurements as products. These products however average day-to-day variability and reduce the amplitude of seasonal oscillations. There are a number of areas where 8-day composites are not sufficient [101], [102], and even 30-day aggregates can fail in particularly cloudy regions or periods. Furthermore, aggregates are biased towards clear-sky values. This, for example, overestimates growing-degree days when 16-day averages are used instead of daily values [103], or it can lead to errors when estimating the phase and amplitude of seasonal oscillations [102].

There is an advantage in analyzing the raw data directly and in estimating the value of missing measurements. The extraction of Fourier components was shown to be more precise when missing measurements where linearly interpolated using adjacent measurements in time, on a per pixel basis, than using the 8-day aggregated data [102]. This is distinct from the completion of a map using spatially adjacent pixels, for the same time period. Spatial interpolation of the missing values, using volumetric splines with altitude as a covariate, was recently shown to be effective at estimating the average temperature in the Italian Alps [101]. By comparison, our algorithm estimates missing pixel values using both temporal and spatial data. It extracts the seasonal variations in land temperature without requiring interpolation of missing pixels, which improves the accuracy of the Fourier analysis when large missing regions are present. The seasonal variations are extracted independently for each pixel, thus including the effect of local variables like altitude, land cover type, soil content, and exposure.

In order to improve the Kriging prediction, spatial information and additional covariates (e.g., land cover type, cloud cover, altitude, distance from the ocean, or interpolated air temperature) could be integrated [104]. With the large amount of spatial data involved, spatial Kriging can become a rate limiting step, but one approach to this addressing this problem would be to sample only a fraction of the valid measurements [101]. However, extended areas with persistent cloud cover are likely to remain a problem for remote sensing data completion algorithms.

### Rainfall

Interpolation of weather station rainfalls reduces the inherent temporal variability and effectively integrates over the natural spatial variability of rainfall [105]. By comparison, the RFE 2.0 remote sensing rainfall estimator can capture the inherent spatial and temporal inhomogeneity of precipitation better. This variability of rainfall has important effects in modeling vector population. During the vector life cycle, large rainfall can kill a large number of larvae, and long periods without rain can eliminate much of the rain fed habitat which can then take a longer time to growth, when starting at a lower set point, than if the rainfall had stayed medium.

The RFE 2.0 product is however limited in its ability to capture warm cloud precipitation, e.g., orographic (relief) precipitation, due to the nature of the satellite measurements used. Depending on the complexity of the terrain, this satellite product may systematically underestimate the amount of rainfall. Orographic effects are however inherently difficult to capture and they represent a topic of ongoing research [64], [65], [106], [107]. For example, neither the standard GPCC [89] nor the CRU 2.1 [88] interpolated rainfall data sets capture relief-related precipitation patterns. One possible improvement to this data set would be to try to capture warm cloud precipitation related to topography by using the PRISM method which was devised to improve weather station interpolation [106]. Alternatively, some of the orographic effects could be captured by using a large number of weather stations as well as latitude and altitude as interpolation covariates [64].

## Conclusions

We have presented robust algorithms to construct an environmental data set where weather data availability is most limited, i.e., in Africa. The air temperature and relative humidity data were constructed using statistical interpolation techniques in order to quantify the precision of each component, an uncommon feature of environmental data sets which allows the user to propagate input uncertainty through their calculations.

Our data set was designed to capture important aspects of the variability of climate. The daily temporal resolution and the kilometer-scale of our air temperature, humidity, rainfall, and land temperature time series, for example, captures both multi-year variability as well as the spatial correlation of climate. In the context of dynamic vector disease modeling, accuracy in input variability leads to, e.g., more realistic estimates of risk to an eradication campaign.

In addition, the land temperature data completion algorithm can be used for other non-climate related periodic data sets. Possible avenues to extend this work would be to study the use of remote sensing products to increase the spatial resolution and the accuracy of our air temperature and relative humidity data sets. Additionally, the land temperature spatial interpolation method could be improved by sampling the measurements neighboring missing regions in order to reduce the amount of computation needed to perform Kriging.

## Supporting Information

File S1 **Supporting information and figures.**
(DOCX)
