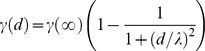# Correction: An Environmental Data Set for Vector-Borne Disease Modeling and Epidemiology

**DOI:** 10.1371/journal.pone.0103922

**Published:** 2014-07-24

**Authors:** The PLOS ONE Staff

In the Methods section, formula M1 is represented incorrectly. The correct formula, provided by the Authors, is shown below.