# Supplementary material for: An Environmental Data Set for Vector-Borne Disease Modeling and Epidemiology
Source: PLoS One. 2014 Apr 22;9(4):e94741. doi: 10.1371/journal.pone.0094741 (PMC3995884; doi:10.1371/journal.pone.0094741)

# Supporting Information

## Fourier decomposition of a signal with missing data

The Fourier decomposition of a signal with missing sections cannot be done using the canonical set of base vectors; the base frequency and its harmonics are not exactly orthogonal when there are missing measurements. However, by identifying the most important Fourier components of the signal it is possible to re-orthogonalize the set of basis vectors relative to one another, and eventually construct a set of orthogonal, Fourier-like vectors, upon which the original signal can be decomposed and then reconstructed.

First, we compute normalized Fourier vectors over the time span of the data for a set of characteristic frequencies. We refer to this set as the descriptor set:

On a data set with missing values, these vectors are not necessarily orthogonal, that is their overlap on the subset of points where the signal has a value is not necessarily zero (the overlap is)$\sum_{signal has value} v_{m}\left( t \right)w_{n}\left( t \right)$. It is important to note that while the sum in the overlap calculation includes only the positions where the signal to decompose has a value, the vectors themselves have a value for all points. N is the number of valid values within the signal, so that its L_2_ norm over the valid values equals to unity. Similarly *c_m_* and *d_n_* are normalization constants which insure that the L_2_ norm of their corresponding vector, over the valid values, equals to unity.

We decompose the signal over these vectors in order of overlap, from largest overlap to small overlap, starting with the constant component (the first vector is always chosen to be v_0_).

Decomposing the data along the descriptor set:

1. Calculate overlap of Fourier vectors remaining in the descriptor set
2. Choose the vector with the largest overlap over the signal, remove it from the descriptor set.
   1. Tabulate this vector, along with its overlap with the data in a separate table
3. Remove the component of the vectors remaining in the descriptor set along the vector which was just removed from the descriptor set.
4. Normalize the remaining vectors in the set.
   1. If a vector’s renormalization factor is greater than 10, remove it from the descriptor set for this weather station. That vector is not sufficiently orthogonal to the current vector to be retained.
5. Return to step 1 unless all the vectors are exhausted or if the signal becomes too low.

The signal along the descriptor set is then simply reconstructed by summing the retained vectors multiplied by their overlap. The reconstructed signal has values for all times, unlike the initial signal. This is a type of interpolation using the periodic components of a signal.

## 2. Kriging prediction details

Detail of the cross-validation for air temperature, dew point, and relative humidity are presented, by weather station. For each weather station, a two-dimensional histogram showing the frequency of either a (measurement, prediction) value pair, or a (measurement, prediction error) value pair is shown. Air temperature histograms are shown in Figures S1 and S2, dew point histograms are shown in Figures S3 and S4, and relative humidity histograms are shown in S5 and S6.

For the (measurement, prediction) value pairs, the diagonal represents accurate prediction: the further from the diagonal the central mass of pairs is, the less accurate the prediction, and the scatter of the pairs an indicator of precision: precise predictions have small scatter, imprecise predictions have large scatter.

By showing the (measurement, prediction error) pairs, we can evaluate if there are systematical changes in prediction errors due to seasonality, or strongly asymmetric error distributions which could under- or over-estimate the interquartile ranges shown in Figs. 10 and 11.

**FIGURE S1: Cross-validation of air temperature, prediction.** Comparison of predicted air temperature value obtained with Kriging and the true value measured at the weather station. Data represented span the 1973-2010 period. For each weather station (as numbered on Figure 10b), we present the frequency of a given (true value, predicted value) pair on a color scale. The blue diagonal line represents theoretically perfectly accurate predictions. Biased predictions will be shifted below or above this diagonal, imprecise estimates appear as a broad scatter.

**FIGURE S2: Cross-validation of air temperature, error.** Computed prediction error, as a function of the true air temperature value measured at the weather station. Data represented span the 1973-2010 period. For each weather station (as numbered on Figure 10b), we present the frequency of a given (true value, predicted error) pair on a color scale. Seasonality in the prediction error will appear as a relationship between predicted value and prediction error.

**FIGURE S3: Cross-validation of dew point, prediction.** Comparison of predicted dew point value obtained with Kriging and the true value measured at the weather station. Data represented span the 1973-2010 period. For each weather station (as numbered on Figure 10b), we present the frequency of a given (true value, predicted value) pair on a color scale. The blue diagonal line represents theoretically perfectly accurate predictions. Biased predictions will be shifted below or above this diagonal, imprecise estimates appear as a broad scatter.

**FIGURE S4: Cross-validation of dew point, error.** Computed prediction error, as a function of the true dew point value measured at the weather station. Data represented span the 1973-2010 period. For each weather station (as numbered on Figure 10b), we present the frequency of a given (true value, predicted error) pair on a color scale. Seasonality in the prediction error will appear as a relationship between predicted value and prediction error.

**FIGURE S5: Cross-validation of relative humidity, prediction.** Comparison of predicted relative humidity value obtained with Kriging and the true value measured at the weather station. Data represented span the 1973-2010 period. For each weather station (as numbered on Figure 10b), we present the frequency of a given (true value, predicted value) pair on a color scale. The blue diagonal line represents theoretically perfectly accurate predictions. Biased predictions will be shifted below or above this diagonal, imprecise estimates appear as a broad scatter.

**FIGURE S6: Cross-validation of relative humidity, error.** Computed prediction error, as a function of the true relative humidity value measured at the weather station. Data represented span the 1973-2010 period. For each weather station (as numbered on Figure 10b), we present the frequency of a given (true value, predicted error) pair on a color scale. Seasonality in the prediction error will appear as a relationship between predicted value and prediction error.


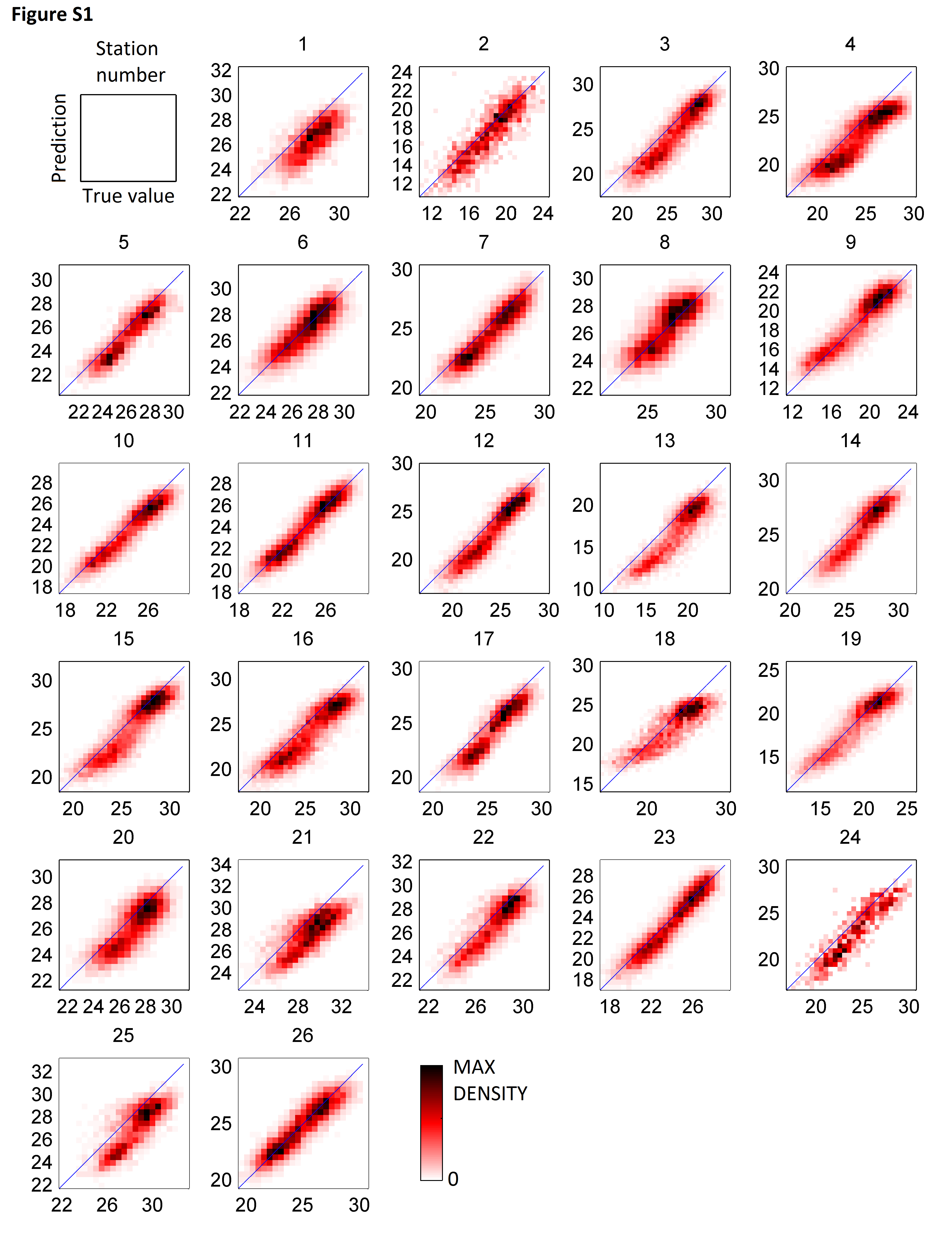

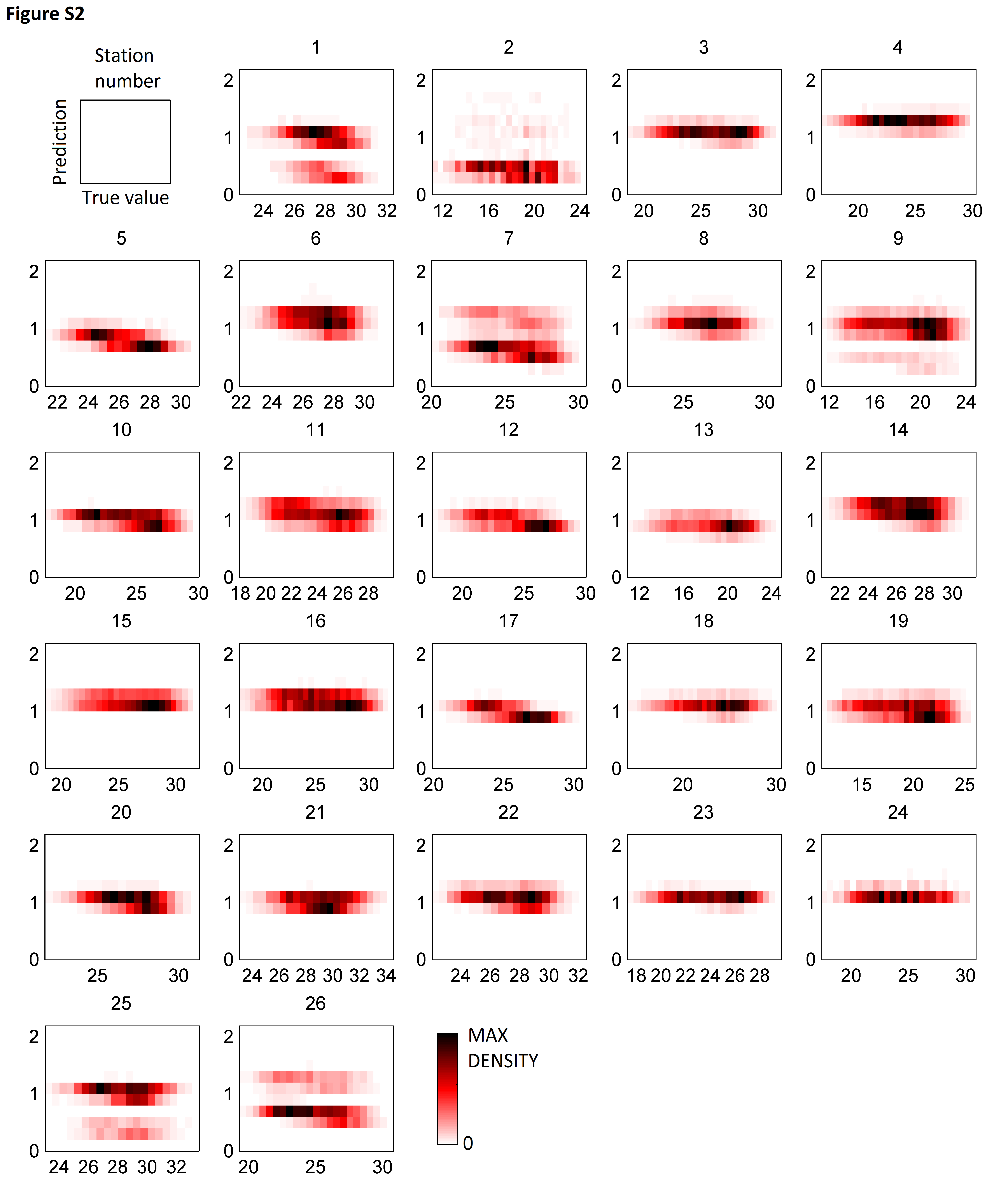

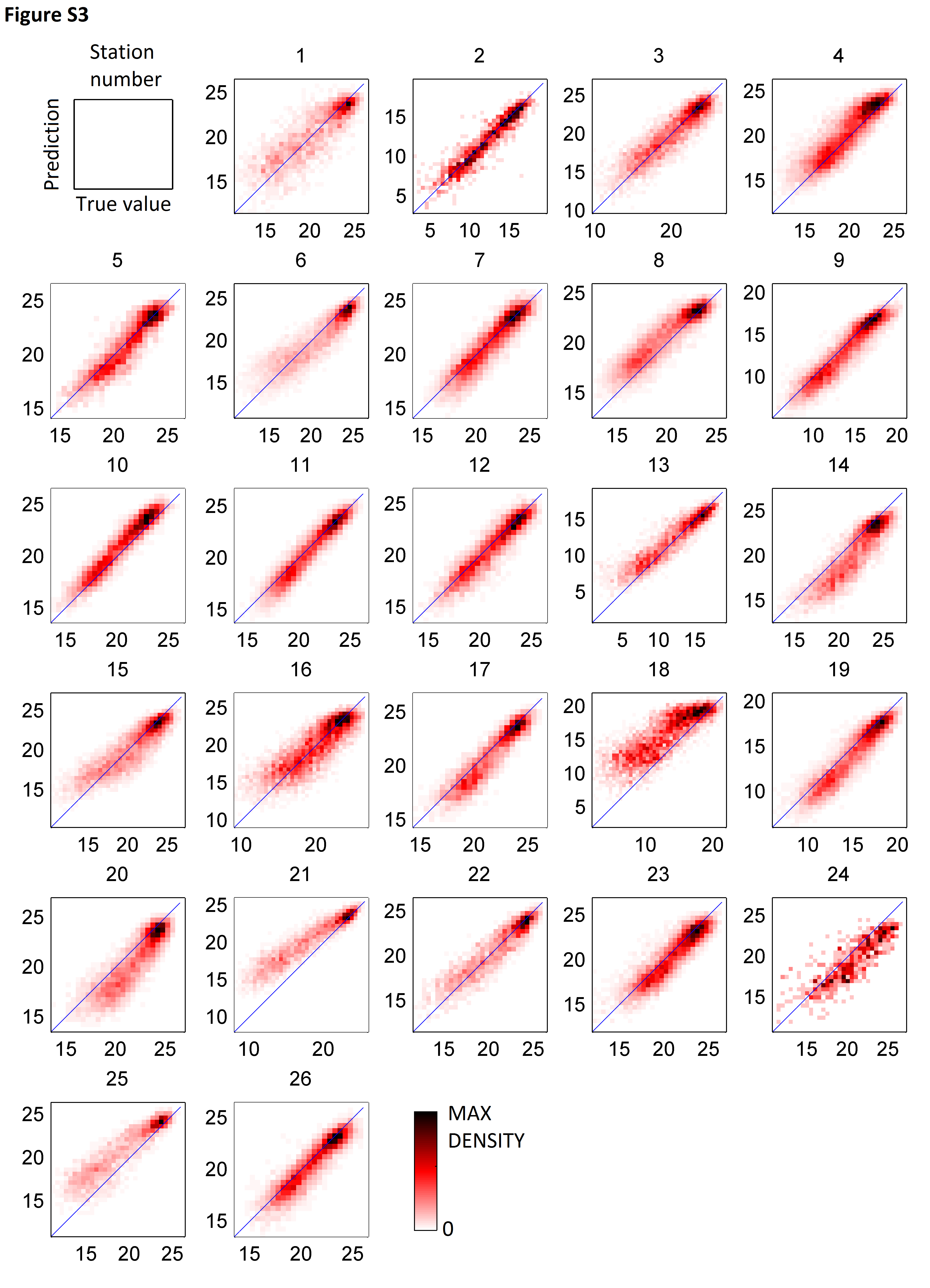

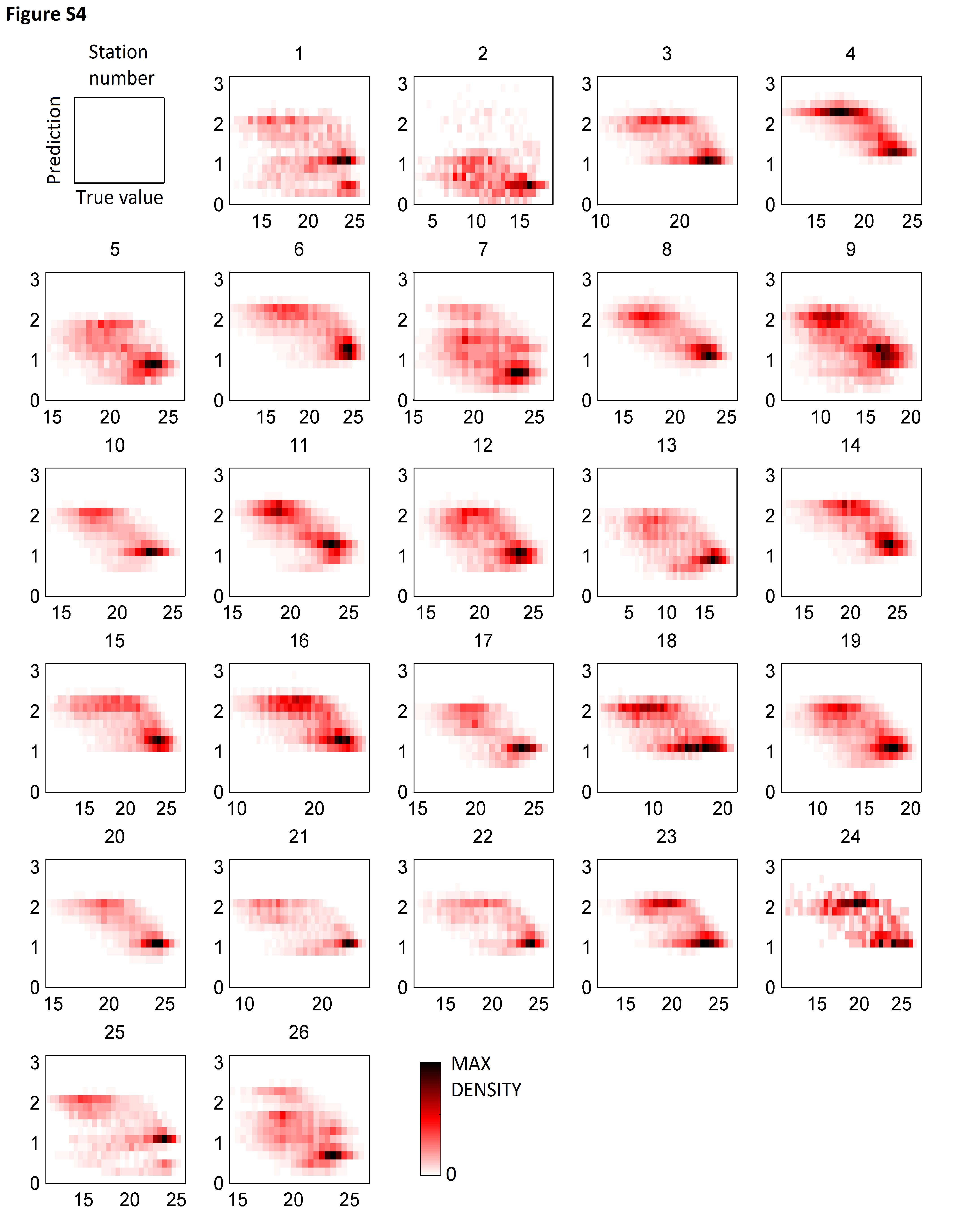

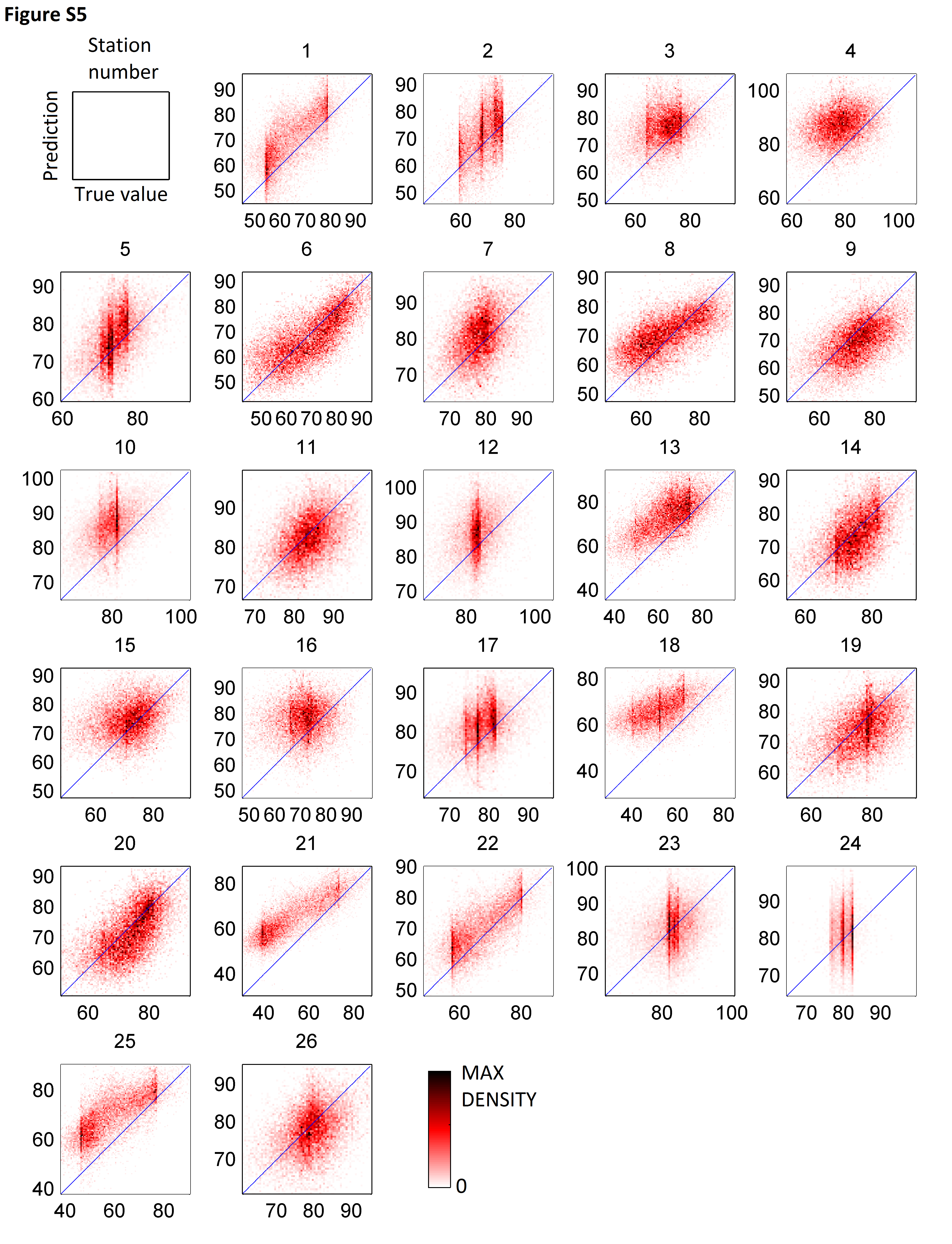

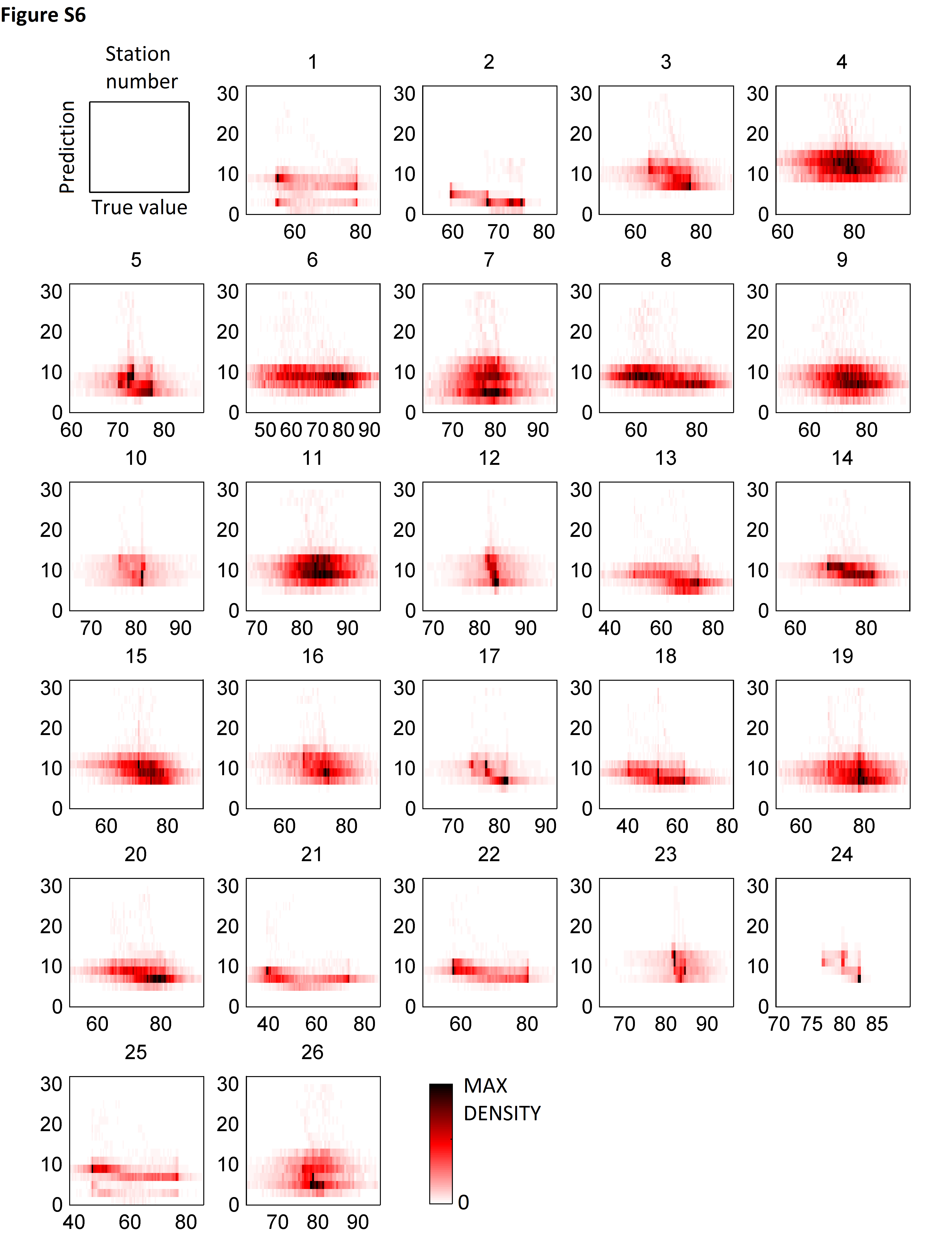

Supplement: File S1 — Supporting information and figures. (DOCX) [file pone.0094741.s001.docx]
